# Concentrated Oxygen

**Published:** 1884-05

**Authors:** 


### CONCENTRATED OXYGEN.

THE curious statement is made by a writer in the London daily "News," who has been collecting some particulars regarding diving operations on the Thames and elsewhere, that men have grown old in this particular business; though in early life they had been in danger of consumption. The theory is a simple one. Men under water in a diving dress breathe a condensed atmosphere, and are therefore inhaling oxygen in a concentrated form; and this, it is assumed, is conducive to the cure of consumption.

### GLENN'S SULPHUR SOAP.

In the cure of skin disease, the local use of sulphur is the best and surest mode of using it, for in that way it comes directly in contact with the skin, which is a wonderful absorbent of sulphur. All such diseases as eczema, tetter, ringworm, litchen, acne, pimples, blackheads, rough and dry scaly skin diseases, must fade before its use like snow in the sunshine. But the question arises, Why can't I use sulphur ointment? No, no: by no means. But you can use Glenn's Sulphur Soap in baths,.and as a toilet soap, daily; and your skin will soon be clear as an infant's, and no one would be able to tell that you ever had a skin complaint.

